# A chemokine gene expression signature derived from meta-analysis predicts the pathogenicity of viral respiratory infections

**DOI:** 10.1186/1752-0509-5-202

**Published:** 2011-12-22

**Authors:** Stewart T Chang, Nicolas Tchitchek, Debashis Ghosh, Arndt Benecke, Michael G Katze

**Affiliations:** 1 Department of Microbiology, University of Washington, Seattle WA, USA; 2 Institut des Hautes Etudes Scientifiques, Bures-sur-Yvette, France; 3 Department of Statistics, Pennsylvania State University, University Park PA, USA; 4 Washington National Primate Research Center, Seattle WA, USA

## Abstract

**Background:**

During respiratory viral infections host injury occurs due in part to inappropriate host responses. In this study we sought to uncover the host transcriptional responses underlying differences between high- and low-pathogenic infections.

**Results:**

From a compendium of 12 studies that included responses to influenza A subtype H5N1, reconstructed 1918 influenza A virus, and SARS coronavirus, we used meta-analysis to derive multiple gene expression signatures. We compared these signatures by their capacity to segregate biological conditions by pathogenicity and predict pathogenicity in a test data set. The highest-performing signature was expressed as a continuum in low-, medium-, and high-pathogenicity samples, suggesting a direct, analog relationship between expression and pathogenicity. This signature comprised 57 genes including a subnetwork of chemokines, implicating dysregulated cell recruitment in injury.

**Conclusions:**

Highly pathogenic viruses elicit expression of many of the same key genes as lower pathogenic viruses but to a higher degree. This increased degree of expression may result in the uncontrolled co-localization of inflammatory cell types and lead to irreversible host damage.

## Background

The threat of a highly lethal viral pandemic remains large in the 21st century. In 2003 SARS-coronavirus (CoV) appeared in Asia and then spread globally, causing greater than 40% mortality in individuals over 60 years of age [1]. Since 1997 highly pathogenic avian influenza, influenza A subtype H5N1, has resulted in high mortality rates (between 33% and 100% depending on the population) [2]. Finally in 2009 swine-origin influenza virus A (SOIV) subtype H1N1 emerged in the Americas and led to a pandemic. As Neumann et al. have observed, this virus shares many characteristics with 1918 influenza which resulted in an estimated 50 million deaths [3]. Furthermore, as Ilyushina et al. have shown, SOIV may mutate into more pathogenic forms in as little as ten passages in cell culture [4].

Injury to the host during respiratory viral infections such as influenza now appears to be the result of inappropriate host responses [5]. Deriving gene expression signatures of high pathogenicity that are robust to biological and experimental variation would be immensely valuable, both in the understanding of pathogenicity as well as in the surveillance of emerging infections. We define a signature as the minimum number of biological variables (here, expressed genes) required to (a) discriminate the phenotype of interest from other phenotypes, (b) identify replicates of the same phenotype, and (c) provide cohesive information about the underlying biological complexity [6]. However, the derivation of gene expression signatures from multiple, independent studies is hindered by a number of factors including the absence of standard experimental protocols, biological variability, and limited sample sizes. Meta-analysis, the analysis of independent but related studies, offers one strategy for overcoming these obstacles.

What host responses are the hallmarks of high pathogenicity? To approach this question we assembled a compendium of host transcriptional responses to low-, medium-, and high-pathogenic infections (which we designate as LPIs, MPIs, and HPIs, respectively) and applied a battery of meta-analysis techniques. Using these techniques we identified signatures of either a digital nature (oppositely expressed in LPIs and HPIs with respect to mock infections) or an analog nature (expressed in a continuum from LPIs to HPIs). We then compared these signatures on the basis of their capacity to predict the outcome of an independent transcriptome experiment. The most accurate signature was analog in nature and implicated excessive chemokine expression and cell recruitment in the development of lethal respiratory infections.

## Results

### Compendium assembly and signature derivation

**Figure 1:**
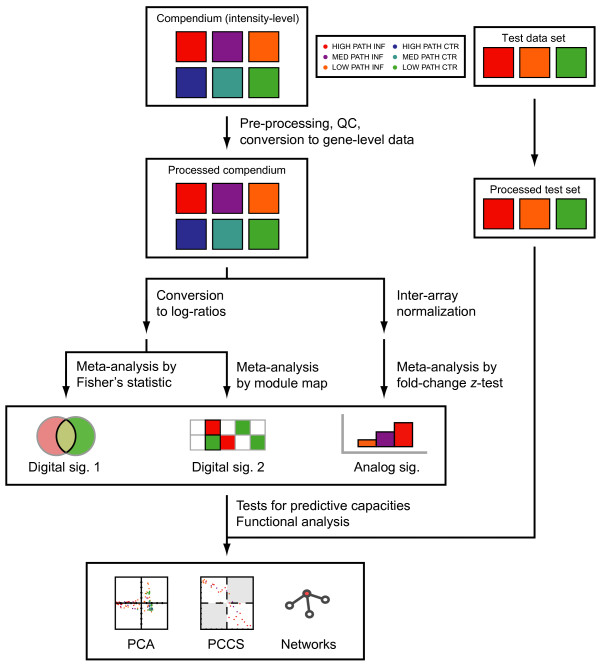
**Meta-analysis workflow**. Briefly the compendium was assembled from multiple microarray data sets and either separated into intensity-level measurements or converted to log-ratios of measurements in infected samples to their respective mock infections. Different meta-analysis methods were applied and resulted in two classes of signatures. Various criteria were used to evaluate signatures. "INF" refers to infected samples, "CTR" to matching mock-infected control samples, "PCA" to principal components analysis, and "PCCS" to Pearson correlation coefficient scatter plot.

We assembled a compendium of microarray data from 12 studies that measured host transcription in mouse lungs following infection with respiratory viruses (Table 1). Six of these studies contained experiments where 100% of the animals succumbed to infection; these involved influenza A subtype H5N1 ("avian influenza"), reconstructed 1918 influenza A, or SARS-CoV strain icHC/SZ/61/03 (Table 1). After merging technical replicates the compendium comprised 733 individual transcriptome measurements with similar numbers of arrays associated with LPIs, MPIs, and HPIs (Figure 1). Data were quality-controlled, further pre-processed, and converted to gene-level data.

**Table 1:** Data sets included in the host response compendium

Virus	Virus strain	Mouse strain	Time points*	Outcome	Reference
Influenza A H1N1	r1918, 2:6 r1918 (1918 HA, NA in Tx91), 5:3 r1918 (1918 HA, NA, M, NP, S in Tx91), Tx91 (A/Texas/36/91)	BALB/c	1, 3, 5 dpi	0% survival (with r1918, 2:6 r1918, 5:3 r1918), 100% survival (with Tx91)	Kash 2006 [30], Tumpey 2005 (for outcome data)[31]
Influenza A H1N1	r1918, PR8	C57BL/6	1, 3, 5 dpi	0% survival (with r1918), 65% survival (with PR8)	Goodman 2009 [32]
Influenza A H3N2	HKx31 (A/HKx31)	BABL/c, C57BL/6	30 hpi	45% survival (in BALB/c), 80% survival (in C57BL/6)	Ding 2008 [33], Toth 1995 (for outcome data)[34]
Influenza A H5N1	HK213 (A/Hong Kong/213/03)	C57BL/6, DBA/2J,	3 dpi	0% survival (in DBA/2J), 30% survival (in C57BL/6)	Boon 2009 [35]
Influenza A H5N1	HK483 (A/HK/483/97), HK486 (A/HK/486/97), HK486PB2MT (A/HK/486 PB2-627K/97)	BALB/c	2, 4 dpi	0% survival (with HK483, HK486PB2MT), 25% (with HK486)	Fornek 2009 [36]
Influenza A H5N1	VN/1203 (Vietnam/1203/04), r1918 (H1N1)	SvEv129	1, 3, 4 dpi	0% survival (with VN/1203, r1918)	Cilloniz 2010 [37]
Mengovirus	vMC0 (UV-inactivated mengovirus)	BALB/c	18 hpi	100% survival	Rosenthal unpub.
RSV	A2	BALB/c	1, 3 dpi	100% survival	Janssen 2007 [38]
RSV	A2	B6:129PF1/J	1 dpi	100% survival	Minor 2010 [39]
SARS-CoV	Urbani	BALB/c (young, aged)	1, 2, 5, 7 dpi	100% survival (in young, aged)	Baas 2008 [40]
SARS-CoV	icUrbani, icGZ02, icHC/SZ/61/03	BALB/c AnNHsd (young, aged)	12 h, 1, 3 dpi	0% survival (in aged with icHC/SZ/61/03), 40% survival (in aged with icGZ02), 100% survival (in young with all 3 virus strains, in aged with Urbani)	Rockx 2009 [41]
SARS-CoV	MA15	129	2, 5, 9 dpi	100% survival	Zornetzer 2010 [42]

Influenza A H1N1	A/CA/04/2009, MA1 (a mouse-adapted strain of A/CA/04/2009)	BALB/c	1, 3, 5 dpi	0% survival (with MA1), 100% survival (with A/CA/04/2009)	Josset in preparation

* Note "dpi" refers to days post-infection. Other annotations are stated as in primary publications. The last row (below line) refers to a data set external to the compendium that was used as a test set for predictions.

We then applied three different methods of meta-analysis to derive gene signatures from the compendium. Hierarchical clustering of the log-ratio data showed that two clusters of genes differed in expression with respect to each other and with respect to HPI vs. LPI (Additional File 1, Figure. S1). This suggested that gene signatures could be identified on the basis of opposite directionality with respect to mock infections. To identify these signatures more rigorously we applied two methods of meta-analysis. The first signature comprised 74 genes identified on the basis of statistical tests for differential expression in each biological condition whose results were combined by Fisher's summary statistic (Additional File 2, Figure. S2A; gene list as Additional File 3, Table S1). The second signature comprised 265 genes identified as a module by similar patterns of co-expression and annotation co-membership (Additional File 2, Figure. S2B, S2C; gene list as Additional File 4, Table S2). Due to their opposite directionality in different pathogenicities, we referred to these signatures as digital in nature. A third signature comprised 57 genes and was derived from the direct comparison of expression in HPIs vs. LPIs without reference to mock infections. Additional normalization allowed inter-array comparisons, and a fold-change-based *z*-test was used to determine differential expression (gene list as Additional File 5, Table S3). Because these genes varied directly between pathogenicities, we referred to this signature as analog in nature. Limited degrees of overlap were observed among the different signatures indicating that the three methods identified different features in the data.

### Capacity of analog and digital signatures to classify host transcription

**Figure 2:**
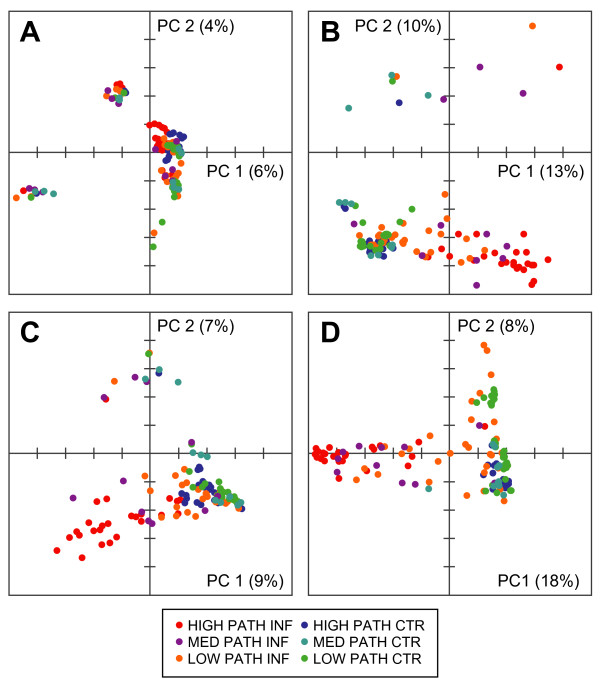
**Separation of biological conditions as determined by PCA in the native compendium (all genes) vs. different gene signature subsets**. Clustering and separation of samples along PC1 as well as percentage variance explained by PC1 (in parentheses) were used as indicators of signature effectiveness. (A) PCA of native compendium (all genes). (B) PCA of Fisher's summary statistic-derived digital signature profiles. (C) PCA of module map-derived digital signature profiles. (D) PCA of *z*-test-derived analog signature profiles.

We initially compared the signatures on the basis of their capacity to structure the biological conditions in the compendium. The expression of each signature set of genes was analyzed by principal components analysis (PCA), and the degree to which LPI-, MPI-, and HPI-associated conditions clustered proximally to conditions of the same pathogenicity and distally from conditions of other pathogenicities was compared. We also compared the ordering and overall separation of the pathogenicity clusters, both by visual inspection and by percentage variance explained. We reasoned that a more effective signature would result in greater separation of pathogenicities oriented along a dimension that also explained a higher proportion of the total variance [7]. Using all the genes in the compendium as input for PCA (i.e., no signatures), the native compendium showed little apparent structure (Figure 2A). Conditions did not discretely cluster by pathogenicity, nor did pathogenicities separate along either first or second principal components (PC1 or PC2, respectively). Using the digital gene signatures, more structure became apparent (Figure 2B, C). Conditions were ordered by severity of pathogenicity along PC1, and the total variance explained by PC1 ranged from 9% to 13% (for the module map- and Fisher's statistic-derived signatures, respectively; *x*-axis in Figure 2B, C). However, the highest degree of clustering and separation was obtained using the analog signature. Conditions clustered more tightly by pathogenicity (particularly in the case of HPI) using this signature than other signatures, and PC1 accounted for a greater proportion of the total variance, 18% (Figure 2D). PCA therefore indicated that the analog signature resulted in the most effective discrimination of host profiles by pathogenicity. We note that the relatively low amount of variance explained by PC1 in the case of each signature was consistent with the presence of significant heterogeneity in the compendium. This heterogeneity may be attributable to a number of different factors including the variety of viruses, times post-infection, mouse strains, laboratories, and technical platforms represented in the compendium. Therefore, we compared signatures on the basis of the relative increases in variance explained by PC1 which aligned with pathogenicity in each case.

**Figure 3:**
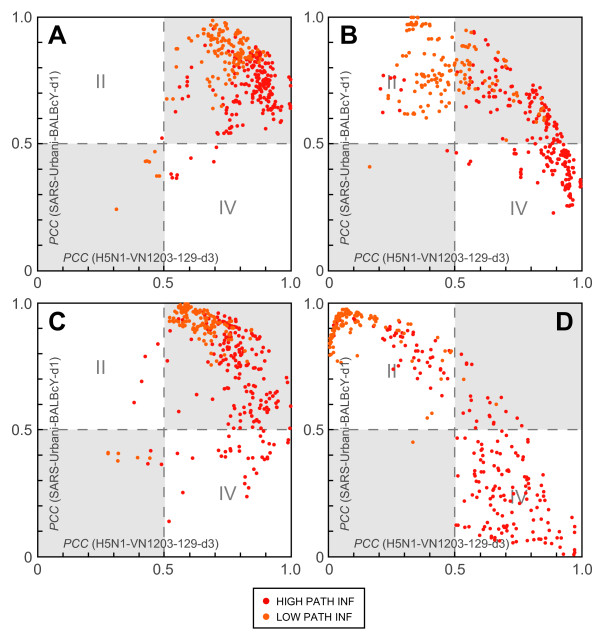
**Correlation of compendium samples to exemplar HPI and LPI transcriptional profiles based on different gene signature subsets**. PCC values of sample profiles to exemplar HPI (*x*-axis) and LPI (*y*-axis) transcriptional profiles were plotted, and quadrants were defined with respect to (0.5, 0.5). LPI and HPI samples located in quadrants II and IV, respectively, were considered accurately classified. (A) Using all genes in the compendium. (B) Using Fisher's statistic-derived digital signature genes. (C) Using module map-derived digital signature genes. (D) Using *z*-test-derived analog signature genes.

We also compared signatures by their capacity to generate sample expression profiles that correlated with other profiles of the same pathogenicity. We reasoned that a more effective signature would result in stronger correlations (higher Pearson correlation coefficients, or PCCs) among similar pathogenicities and weaker correlations (lower PCCs) among dissimilar pathogenicities. On a scatter plot of PCCs to exemplar LPI and HPI profiles, more samples would appear in two quadrants, with more LPI samples in quadrant II (LPI-high/HPI-low) and more HPI samples in quadrant IV (LPI-low/HPI-high) where quadrants are defined by an equal partitioning of the PCC scatter plot plane at (0.5, 0.5). Using all of the genes in the native compendium, LPI and HPI samples appeared primarily in one quadrant, high for both LPI and HPI (Figure 3A). This indicated that LPI and HPI transcriptional profiles were correlated overall (for the majority of genes) and that LPI and HPI samples separated poorly from each other on the basis of overall transcriptional profiles. Digital gene signatures resulted in improved segregation of samples by pathogenicity, as indicated by increased numbers of LPI and HPI samples in quadrants II and IV, respectively (white regions in Figure 3B, C). However, the analog signature resulted in the largest overall segregation of biological samples, with the highest number of LPI and HPI samples appearing in opposite quadrants (Figure 3D). Therefore PCA and PCC scatter plots were consistent in indicating that the analog signature achieved the best separation of transcriptional profiles by pathogenicity.

### Capacity of the analog signature to predict test set pathogenicity

**Figure 4:**
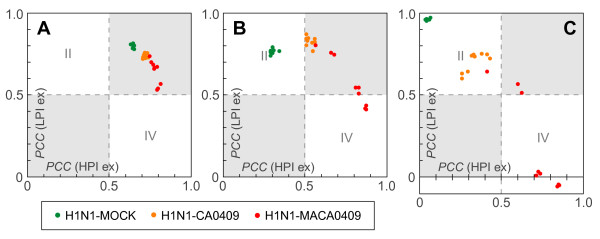
**Prediction of test set classification by correlation to exemplar HPI and LPI transcriptional profiles**. The same exemplar samples were used for HPI (*x*-axis) and LPI (*y*-axis) as in Figure. 3, and quadrants were similarly defined. PCC values of test set samples to each exemplar were plotted, and LPI and HPI samples located in quadrants II and IV, respectively, were considered accurately classified. (A) Using all genes in the compendium. (B) Using Fisher's statistic-derived digital signature genes. (C) Using *z*-test-derived analog signature genes.

We then tested the capacity of each signature to predict the pathogenicity of a data set external to the compendium. Specifically we utilized a microarray data set measuring transcription in the lungs of mice infected with either swine-origin influenza A virus (SOIV) subtype H1N1 strain CA/04 (non-lethal in mice) or the mouse-adapted variant MA1 CA/04 (lethal in mice) at days 1, 3, and 5 post-infection (Josset et al., in preparation). PCC scatter plots were generated using the test set samples and the same LPI and HPI exemplars as above (in Figure 3). We again reasoned that the most effective gene signature would yield the best opposite-quadrant separation of LPI and HPI samples. Comparing the better-scoring digital signature (i.e., generated by Fisher's statistic) and the analog signature, we found that the analog signature yielded the best segregation of test set samples (Figure 4). In particular samples from the lethal variant MA1 CA/04 correlated strongly with the exemplar HPI, indicating that this and other HPIs resulted in similar transcriptional profiles for these genes (Figure 4C). Counting the number of LPI and HPI test set samples appearing in quadrants II and IV, respectively, and considering these as correctly predicted, we found that the analog signature resulted in an accuracy rate of 83% (15 of 18 samples), exceeding the rates obtained using all genes (0 of 18) or the better-scoring digital signature (3 of 18).

### Characterization of the digital and analog signatures and the role of chemokines

Signature genes may in turn identify mechanisms of pathogenesis. To identify functions and pathways dysregulated across HPIs, we analyzed the annotations associated with each set of signature genes. Annotations for the better-performing digital signature (derived from Fisher's statistic) indicated that a number of pathways may have been altered during HPIs. Genes up-regulated in HPI were associated with inflammation, apoptosis, cell signaling, and cell proliferation, while genes down-regulated in HPI were associated with cytochrome P450-related enzymatic activity. Protein-protein interactions linked many of these genes together indicating possible cooperative effects (Additional File 6, Figure. S3A, S3B). Similar annotations were found to be enriched among the genes of the remaining digital signature (derived by module mapping) including cell differentiation, inflammatory response, and chemical homeostasis.

**Figure 5:**
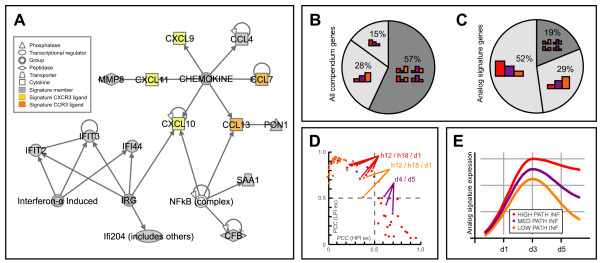
**Characterization of the analog gene signature by known interactions and expression across pathogenicity groups**. (A) Network showing known interactions among a subset of analog signature gene products related to chemokines and interferon-related signalling. Signature genes are shaded with those also encoding CXCR3 ligands in yellow and those also encoding CCR3 ligands in orange. Specific classes of molecules are indicated by shape (legend). (B) Proportion of all genes in the compendium ordered from least to greatest or greatest to least according to pathogenicity as indicated in lightly shaded regions. Genes not matching either of these patterns are counted in darkly shaded regions. (C) Proportion of analog signature genes ordered from least to greatest or greatest to least according to pathogenicity (following same color schema as part B). (D) PCC scatter plot of analog signature genes indicating the time of sample collection. (E) Model of analog signature gene expression during infections of different pathogenicities consistent with observations in this study.

The analog signature, which outperformed both digital signatures in our tests, was significantly enriched in genes associated with the inflammatory response (11 genes, Benjamini-Hochberg-adjusted *p *= 2.7 × 10^-7^) and chemokine activity (6 genes, Benjamini-Hochberg-corrected *p *= 1.9 × 10^-5^). A network showing known interactions among analog signature gene products highlighted cooperativity between the inflammatory/interferon-response pathway and chemokines such as CXCL10 (Figure 5A). This analysis indicated that different gene signatures identified inflammation as dysregulated during HPIs despite limited overlap at the gene level.

Closer examination of the analog signature genes indicated a relationship between signature expression and pathogenicity. We determined the proportion of signature genes whose expression was ordered by pathogenicity (from least to greatest in LPI, MPI, and HPI, or vice versa) and compared this proportion to those expected by chance or observed among all genes. Assuming an equal distribution of genes among pathogenicity groups, we expected 33% to display one of these two patterns by chance. By comparison we observed that 43% of all genes in the compendium displayed these patterns (Figure 5B). However, an even greater proportion of analog signature genes displayed these patterns, 81%, consistent with a correlation between expression and pathogenicity (Figure 5C). Using a binomial distribution we calculated the chance enrichment of genes being proportionally expressed with pathogenicity to be *p *< 0.0019 in the case of Figure 5B (among all compendium genes) and *p *< 6.6 × 10^-23 ^in the case of Figure 5C (among analog signature genes). Furthermore, if we considered the distribution of gene profiles among all compendium genes to be expected, the enrichment of proportionally expressed genes in the analog signature was statistically significant at *p *< 1.6 × 10^-13^.

Analog signature genes expressed in proportion to pathogenicity could in turn be ranked by level of expression in HPIs (Additional File 7, Figure. S4). The most highly expressed signature genes in HPIs were interferon-induced including *IFIT3, CXCL10, IIGP1*, and *IGTP*, consistent with the prominence of these genes in functional and network analyses (Figure 5A).

We also examined the temporal expression dynamics of analog signature genes during infection. More detailed examination of the PCC scatter plot (Figure 3D) showed that early time points during HPIs clustered closely with early time points from LPIs and middle time points from MPIs (Figure 5D). These conditions were in turn intermediate to extrema from other HPI and LPI conditions (i.e., early LPI and late HPI conditions). This suggested that LPI, MPI, and HPI may have activated (or suppressed) the same set of genes but that HPI may have done so more rapidly or to higher levels, resulting in levels of expression not reached during LPI or MPI (Figure 5E).

**Figure 6:**
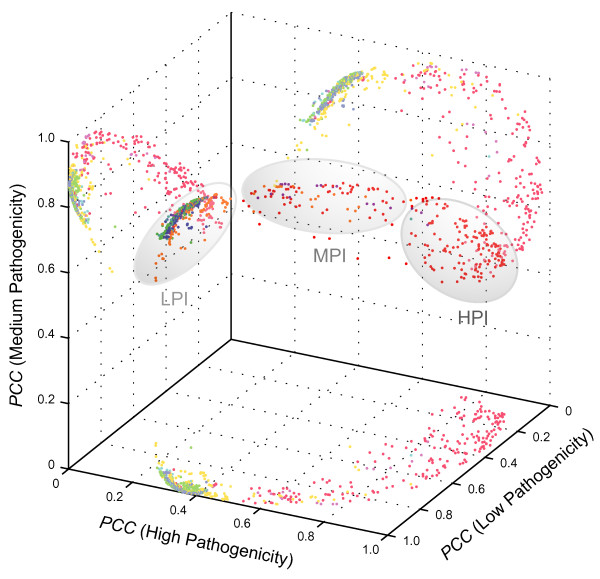
**3D PCC scatter plot showing correlations in analog signature gene expression among LPI, MPI, and HPI pathogenicity groups**. Rather than using a single biological sample as an exemplar of a given pathogenicity (as in Figure. 3), all samples of a given pathogenicity were averaged together (using variance-weighted averages) and each average was used as a new exemplar. Samples (as data points) are colored following the legend in Figure. 1. Dots colored more lightly represent 2D projections of correlations for given pairs of pathogenicity groups.

Finally to gain an overall perspective of the expression of analog signature genes in all pathogenicity groups, we generated a PCC scatter plot that included all samples in the compendium, including MPI-associated samples (Figure 6). New exemplars were generated for each pathogenicity group representing the average expression of signature genes during LPIs, MPIs, and HPIs, and PCCs were calculated to each new exemplar. The resulting scatter plot showed that analog signature genes were expressed similarly within each pathogenicity group (Figure 6). In particular LPI samples correlated highly with each other (i.e., with high PCCs) as did MPI and HPI samples. However, expression also varied continuously among different groups, with expression overlapping between LPI and MPI, as well as between MPI and HPI, consistent with an analog relationship between this signature and pathogenicity.

## Discussion

Experiments on the host responses to infection have generated a wealth of data over the past decade. A number of these have focused on respiratory viruses including several which resulted in lethality in experimental animal models. What distinguished these infections from others where experimental animals recovered was the subject of our analysis. To this end we assembled a compendium of transcriptome measurements from the lungs of mice and used meta-analysis to determine gene signatures that distinguished high- from low-pathogenicity infections. To our knowledge ours is the first meta-analysis study to focus on high-pathogenicity infections (HPIs). Previous studies have applied meta-analysis to infection data but focused on more general features of disease. For example, Jenner and Young analyzed the transcriptomes of various host species (mice, macaques, and humans) infected by bacteria or viruses and identified a signature of approximately 500 genes [8]. This signature showed that TLRs and pathogen-mediated signalling were broadly activated during infection but did not identify features resulting in animal lethality. Likewise Pennings et al. examined the transcriptional profiles of lung inflammation (in mice and macaques) due to various factors (including viruses, bacteria, chemicals, and allergens) and derived a 383-gene signature up-regulated during inflammation [9]. This signature focused on the interferon response and immune signalling, but likewise no correlate of pathogenicity was identified. In addition both studies relied mainly on hierarchical clustering to determine signatures of interest. While hierarchical clustering provides a useful overview of high-dimensional data, it has the disadvantage of lacking a strong statistical basis (i.e., hypothesis testing) and its results (gene clusters) can be difficult to relate to specific outcomes.

In contrast to previous studies, our goal was to determine a gene signature specific for HPIs. We therefore applied an ensemble of meta-analysis methods and derived multiple signatures which we compared by multiple criteria including the capacity to predict the outcome of a test data set. Each method had a different statistical rationale and could be expected to identify different features of the compendium. We had no *a priori *expectation of which signature would produce the best outcome in terms of these criteria.

The 74-gene signature derived using Fisher's summary statistic showed a modest capacity to separate samples into pathogenicity groups and to predict test set pathogenicity. A number of the genes in this signature have known connections to the immune response and the outcome of respiratory infections, e.g., genes for the chemokines osteopontin (*SPP1*) and RANTES (*CCL5*) and the chemokine receptor CCR2. In previous studies mice lacking *SPP1 *and *CCL5 *were found to clear influenza infection with no adverse effects [10,11], while mice lacking *CCR2 *survived infection by a mouse-adapted influenza A virus that killed wild-type mice [12]. These studies suggest that at least some of the genes up-regulated during HPIs are non-essential to resolving influenza infection and that dysregulated activation may even be detrimental to the host.

The best performing signature, however, comprised 57 genes derived using the fold change-based *z*-test. Strikingly the majority of the genes in this signature were expressed at levels that corresponded with pathogenicity. For the majority of these genes, expression was lowest in LPI, highest in HPI, and intermediate in MPI (which had not been used in the derivation of the signature). In this case the signature appeared to provide a continuously variable signal that matched with output, a characteristic of analog signals. This finding also suggests that high- and low-pathogenic infections may result in the expression of the same key genes but with different kinetics. In particular HPIs may result in increased expression of signature gene products beyond the capacity of the host to cope, resulting in irreversible damage.

Genes of the analog signature largely differed from those in the digital signatures but overlapped at the pathway level. For example, chemokine genes were present in both Fisher's statistic-derived and analog signatures. However, the analog signature displayed additional coherence, encoding multiple chemokines for the same receptor, namely MIG (*CXCL9*), IP-10 (*CXCL10*), and I-TAC (*CXCL11*) which all serve as ligands for the receptor CXCR3 [13]. CXCR3 is expressed on the surface of Th1 cells as well as NK and NKT cells and regulates the migration of these cells to sites of infection. Recent evidence indicates that CXCR3 engagement may drive further recruitment and inflammation, resulting in a positive feedback loop that may contribute to pathogenicity [13]. The analog signature also included genes for chemokines that bind the CCR3 receptor, specifically MCP-3 (*CCL7*) and MCP-4 (*CCL13*). CCR3 is the major receptor expressed on eosinophils and has previously been shown to have a role in the promotion of lung inflammation [14]. Interestingly CXCR3 ligands have been postulated to be antagonists for CCR3 [15], and the expression of both sets of chemokines may reflect a high degree of dysregulation during HPIs and the recruitment of multiple immune cell types that may not normally co-localize during a controlled infection.

In addition our signatures also identified genes that were down-regulated during HPI, relative to either mock infection or LPI. For example the Fisher's statistic-derived signature included several genes whose products may normally help to resolve infection. Hepsin (*HPN*) has been found to cleave influenza hemagglutinin directly resulting in non-infectious virus particles [16]. Likewise surfactant-associated protein A1 (*SFTPA1*) and surfactant-associated protein D (*SFTPD*) maintain pulmonary structure but have also been found to inhibit infectivity by mechanisms that remain to be elucidated [17,18]. The analog signature contained a similar set of genes but expressed at lower levels in HPIs compared to LPIs rather than to mock infections. Among these genes were several members of the secretoglobin family including *SCGB1A1, SCGB3A1*, and *SCGB3A3*. Interestingly many secretoglobins are expressed specifically in the lung epithelium and may contribute to lung repair following damage [19,20]. For example, uteroglobin/CC16 (*SCGB1A1*) is secreted by bronchiolar Clara cells and postulated to have a role in reducing airway inflammation, though its exact function remains to be elucidated [21].

## Conclusions

Together these results implicate dysregulated cell recruitment and inflammation (up-regulated in HPI) and impaired lung protection (down-regulated in HPI) in the events leading to lethality in mice. The different meta-analysis methods all aimed to identify pathology-relevant gene expression, and concordance at the pathway level offered a degree of cross-validation. However, our results also demonstrate that the methods identified different features of the data and argue for the application of multiple methods in future studies when the most predictive features of the data are not known in advance.

Finally our finding that pathogenicity corresponds to the expression levels of a defined set of genes may help to inform future therapies. Altering the outcome of infection may not require opposite regulation to be achieved in particular pathways; instead, adequate tempering or delay of those pathways may be sufficient. For example a large number of potential CXCR3 antagonists have been developed recently in the context of other inflammatory diseases [22]. These may provide the basis for novel therapeutic strategies that reduce, but need not completely eliminate, receptor activation to effect control of highly pathogenic respiratory infections.

## Methods

### Data

A literature search for microarray data pertaining to respiratory viral infections in mouse models was performed, and candidate data sets were identified. Data were included in the compendium if they were derived from experiments on wild-type mice, as this ensured normal development. We also required that raw data be measured on whole-genome array platforms. Data were sourced from either in-house or external studies (Table 1). In the latter case, data were downloaded from public repositories or obtained directly from study authors. Pathogenicities of the corresponding experiments were determined by examination of the published outcome data (e.g., mortality or morbidity data or in cases of LPIs, statements attesting to the survival or recovery of all animal subjects). We defined HPIs as experiments resulting in 0% survival, LPIs as experiments resulting in 100% survival, and MPIs as experiments resulting in intermediate levels of survival (between 0% and 100%, non-inclusive). A biological condition was defined as any unique study/time point/virus strain/mouse strain combination.

### Data pre-processing and quality control

Data from Agilent platforms were obtained from Agilent Feature Extraction software and pre-processed in GeneSpring GX 11 (Agilent Technologies, Santa Clara CA) using median-background subtraction on a per-study basis. Data from the Affymetrix platform were pre-processed in GeneSpring GX 11 using the MAS5 algorithm. Quality control was restricted to probe-level filtering. Probes with present flags in all of the technical replicates for at least one biological condition in a given study were retained in the data set for that study. For ratio-based meta-analysis, two-channel data were reformatted as log_2_-ratios (infected/mock-infected), and one-channel data were converted to a two-channel-like format where mock-infection intensities were averaged prior to ratio. For meta-analysis based on direct comparison of intensities, two-channel data were separated into individual channels, and inter-array normalization was performed using NeONORM [23]. Additional data preparation involved converting probe- to gene-level data. Measurements for probes mapping to multiple genes were duplicated for each gene, and probes mapping to the same gene were averaged. RefSeq IDs were chosen as the common gene identifier. Probes not mapping to RefSeq IDs were excluded. Biological and technical replicates were averaged for the intensity-based approaches. Log-ratio measurements from all studies were combined resulting in measurements for 27,567 genes by 397 biological conditions. All data-handling tasks were performed using custom scripts in Perl.

### Fisher's summary-statistic method

We applied the Fisher's summary-statistic method based on the previous application of Rhodes et al. [24]. Our implementation was derived from MADAM (Meta-Analysis Data Aggregation Methods), a toolbox for the R statistical language [25]. Statistical tests were performed on a per-gene basis for all arrays associated with a given biological condition. Specifically log-ratio measurements for each array were re-normalized by dividing by the standard deviation of the array, and one-sided *t*-tests were used to identify genes up- and down-regulated relative to mock infection, i.e., for the null hypotheses *H*_0_: log_2 _(infected/mock) ≤ 0 and *H*_0_: log_2 _(infected/mock) ≥ 0, respectively. *p*-values for each directionality (up- or down-regulation) and pathogenicity (HPI or LPI) were combined according to Fisher's summary statistic *S *= -2 ∑ log *p *[26]. The significance of *S *was determined by a permutation test whereby *p*-values were shuffled 1000 times among biological conditions, *S *was re-derived each time, and the resulting *S *values constituted the null distribution. An FDR-corrected *p*-value of 0.05 was used as a threshold for significance. Genes that were found to be both up- and down-regulated for a given pathogenicity (HPI or LPI) were excluded from further analysis. The signature comprised genes found to be oppositely regulated in HPI and LPI.

### Module map method

The module-mapping method of Segal *et al*. identifies modules of co-expressed genes from microarray data based on co-expression within the compendium and co-membership in previously annotated gene sets [27]. We used the module-map method as implemented in the software package Genomica (available at http://genomica.weizmann.ac.il) using the log-ratio compendium and default settings. Modules were tested for associations with HPI and LPI outcomes, and the signature comprised the largest module found to be oppositely regulated in HPI and LPI.

### Fold change-based z-test

Intensity-based measurements were used to test for differential expression in all HPIs vs. all LPIs. Specifically a fold-change-based *z*-test was used to compute the statistical significance of log_2 _(HPI/LPI) values according to intra- and inter-group variability. Statistically significant log_2 _(HPI/LPI) values were then ranked by size, and genes with log_2 _(HPI/LPI) < -1.6 or > 2 were retained. The cut-offs were selected based on a histogram of fold-changes over all statistically significantly differentially expressed genes.

### Functional and network analysis

We used NIH DAVID (Database for Visualization and Integrated Discovery) and Ingenuity Pathways Analysis (Ingenuity Systems, Redwood City CA) for further analysis of meta-analysis results. DAVID identifies GO terms and other functional annotations enriched in gene lists [28]. An enrichment score is calculated for clusters of similar annotations as -log_10 _of the geometric mean of the hypergeometric test *p*-values within the cluster [29]. We assessed enrichment of the GO categories GOTERM_BP_ALL, GOTERM_CC_ALL, and COTERM_MF_ALL and pathway categories BIOCARTA and KEGG_PATHWAY; otherwise, default options in DAVID were used. We identified significant annotations by enrichment scores > 1.30 representing a mean cluster *p *< 0.05.

## Authors' contributions

STC planned the study, performed the research related to the compendium assembly and processing and digital gene signature derivation, and drafted the manuscript. NT performed the research related to the analog gene signature derivation. DG contributed to the interpretation of the data. AB drafted portions of the manuscript. MGK conceived of the study design and helped to draft the manuscript. All authors read and approved the final manuscript.

## Supplementary Material

Additional file 1 **Figure S1. Hierarchical clustering identifying gene clusters oppositely regulated across various conditions in the compendium**. Shown are log_2_-ratios of intensities in infected to mock-infected samples for genes whose ratios were non-zero across all the measurements in the compendium. The two clusters of interest are boxed in yellow and enumerated to the right of the heat map. Heat maps were generated using the heatmap2 function from the gplots package in R statistical environment with clustering by Euclidean distance and the complete linkage method. Click here for file

Additional file 2 **Figure S2. Derivation of digital gene signatures**. (A) The 74-gene signature comprised 44 and 30 genes derived from the intersection of four parent gene sets: those up-regulated in HPI ∩ down-regulated in LPI and those down-regulated in HPI ∩ up-regulated in LPI. Each parent gene set was derived using Fisher's summary statistic following one-tailed *t*-tests on each biological condition in the compendium. Each intersection was found to represent a significantly larger proportion of its two parent gene sets than expected by chance (as determined by hypergeometric test, *p *< 0.05). (B) Module map resulting from applying Genomica to the log-ratio compendium. Module up-regulation in a given condition is indicated in red, and module down-regulation in green. (C) Expression of Module 5 comprising 265 genes. HPI-associated arrays are indicated in purple, LPI-associated arrays in blue. Values shown are consistent with the overall pattern of module expression in those arrays in which the module is significantly expressed. Module 5 completely subsumed Module 6 and was used in subsequent analysis. Click here for file

Additional file 3 **Table S1. Digital signature genes by Fisher's summary-statistic**. Click here for file

Additional file 4 **Table S2. Digital signature genes by module-mapping**. Click here for file

Additional file 5 **Table S3. Analog signature genes by fold change-based *z*-test**. Click here for file

Additional file 6 **Figure S3. Characterization of the 74-gene digital signature of pathogenicity by networks of known interactions**. Genes present in the signature are indicated in gray shapes. (A) For the 44-gene subset up-regulated in HPIs and down-regulated in LPIs. (B) For the 30-gene subset down-regulated in HPIs and up-regulated in LPIs. Click here for file

Additional file 7 **Figure S4. Expression levels of select analog signature genes in HPI, MPI, and LPI conditions**. These genes met the criterion of being expressed from greatest to least or from least to greatest (inset) by pathogenicity. Error bars represent standard errors of the means across all HPI, MPI, or LPI conditions in the compendium. Click here for file
