# Supplementary figures and images for: A chemokine gene expression signature derived from meta-analysis predicts the pathogenicity of viral respiratory infections
Source: BMC Syst Biol. 2011 Dec 22;5:202. doi: 10.1186/1752-0509-5-202 (PMC3297540; doi:10.1186/1752-0509-5-202)

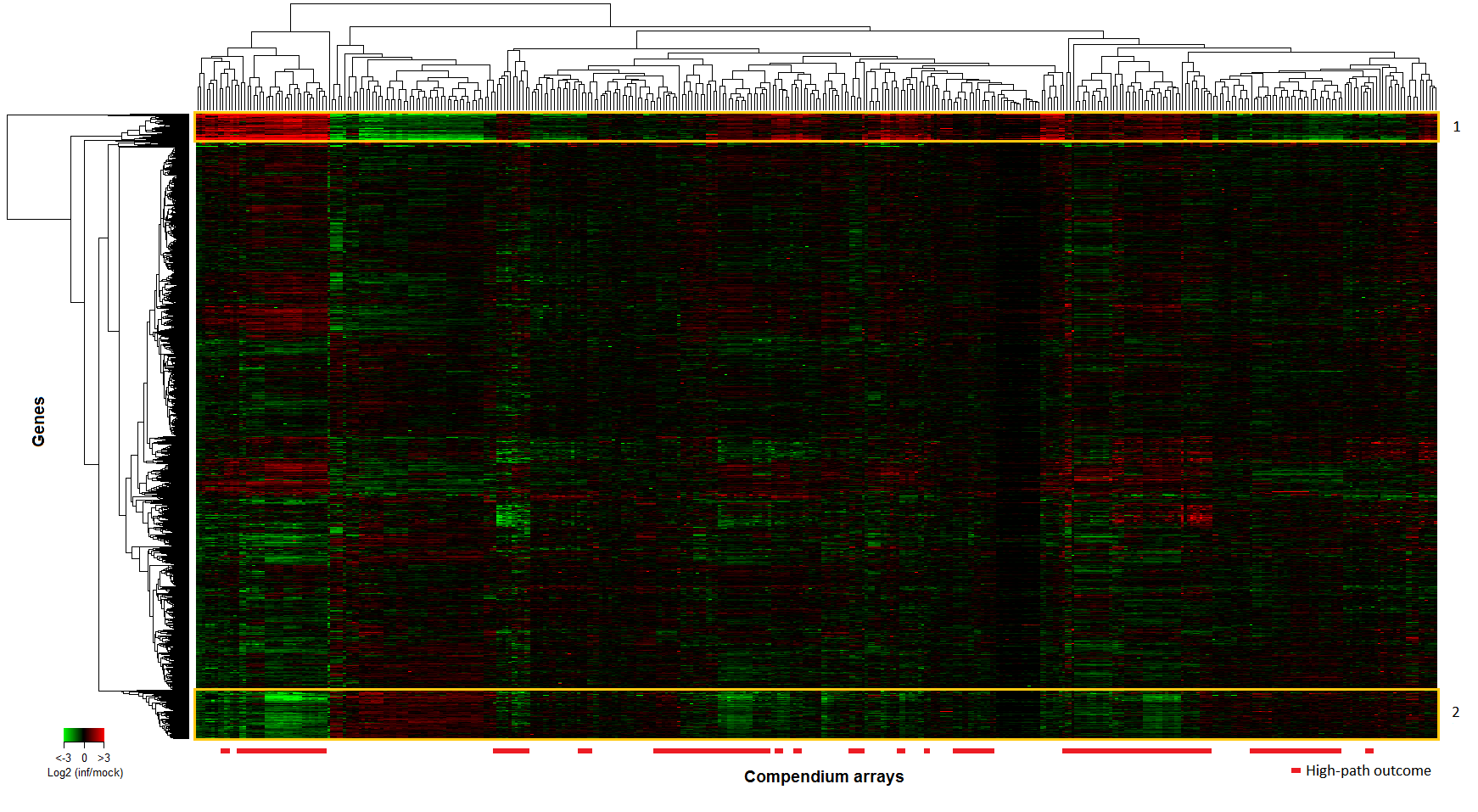

Supplement: Additional file 1 — Figure S1. Hierarchical clustering identifying gene clusters oppositely regulated across various conditions in the compendium. Shown are log2-ratios of intensities in infected to mock-infected samples for genes whose ratios were non-zero across all the measurements in the compendium. The two clusters of interest are boxed in yellow and enumerated to the right of the heat map. Heat maps were generated using the heatmap2 function from the gplots package in R statistical environment with clustering by Euclidean distance and the complete linkage method. [file 1752-0509-5-202-S1.TIFF]

**A**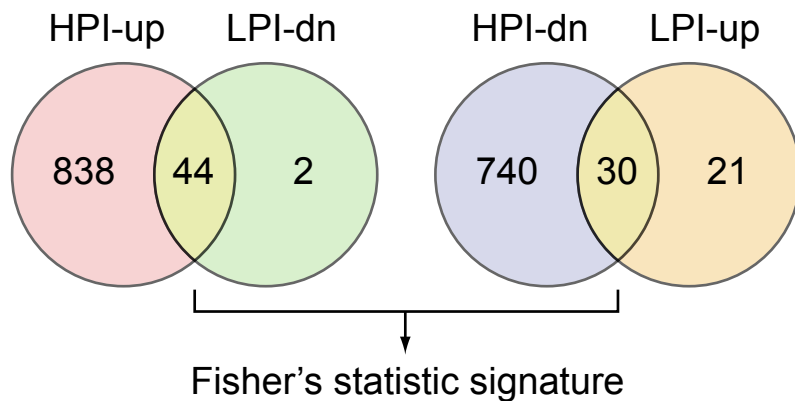**B**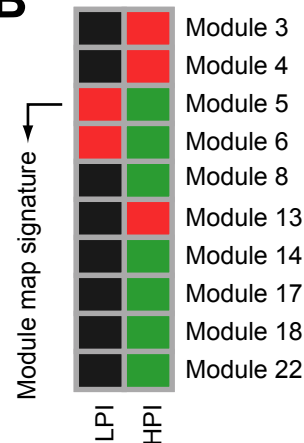**C**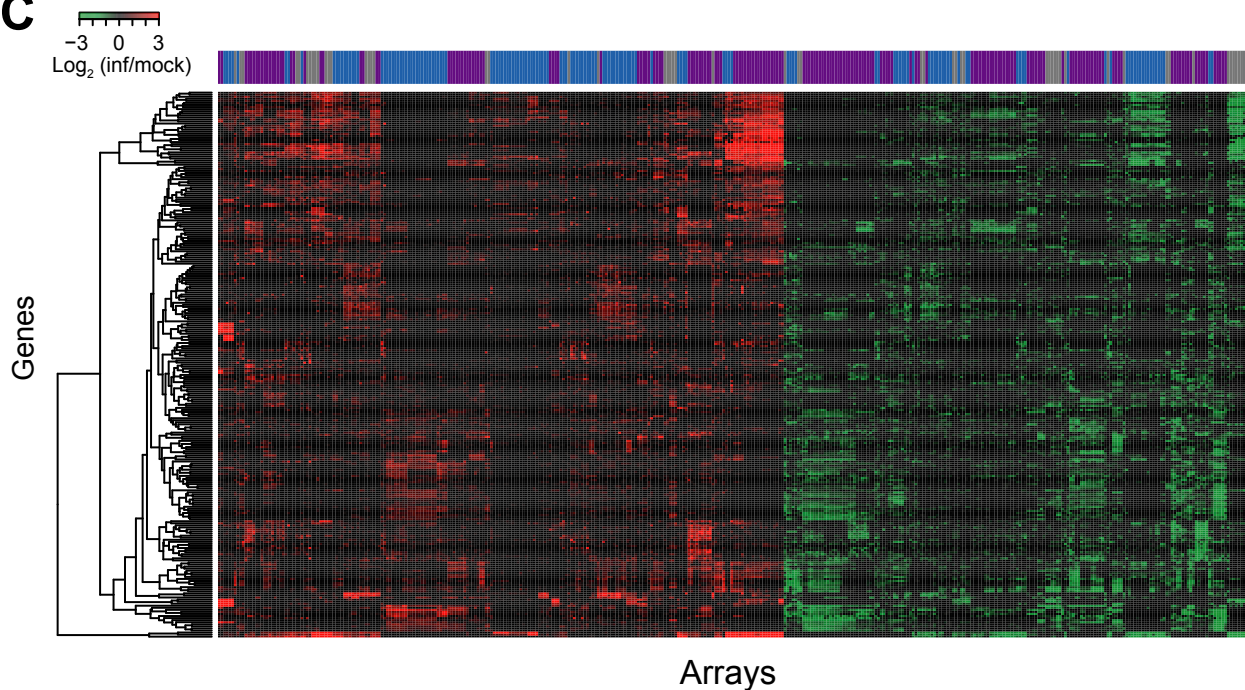

Supplement: Additional file 2 — Figure S2. Derivation of digital gene signatures. (A) The 74-gene signature comprised 44 and 30 genes derived from the intersection of four parent gene sets: those up-regulated in HPI ∩ down-regulated in LPI and those down-regulated in HPI ∩ up-regulated in LPI. Each parent gene set was derived using Fisher's summary statistic following one-tailed t-tests on each biological condition in the compendium. Each intersection was found to represent a significantly larger proportion of its two parent gene sets than expected by chance (as determined by hypergeometric test, p < 0.05). (B) Module map resulting from applying Genomica to the log-ratio compendium. Module up-regulation in a given condition is indicated in red, and module down-regulation in green. (C) Expression of Module 5 comprising 265 genes. HPI-associated arrays are indicated in purple, LPI-associated arrays in blue. Values shown are consistent with the overall pattern of module expression in those arrays in which the module is significantly expressed. Module 5 completely subsumed Module 6 and was used in subsequent analysis. [file 1752-0509-5-202-S2.PDF]

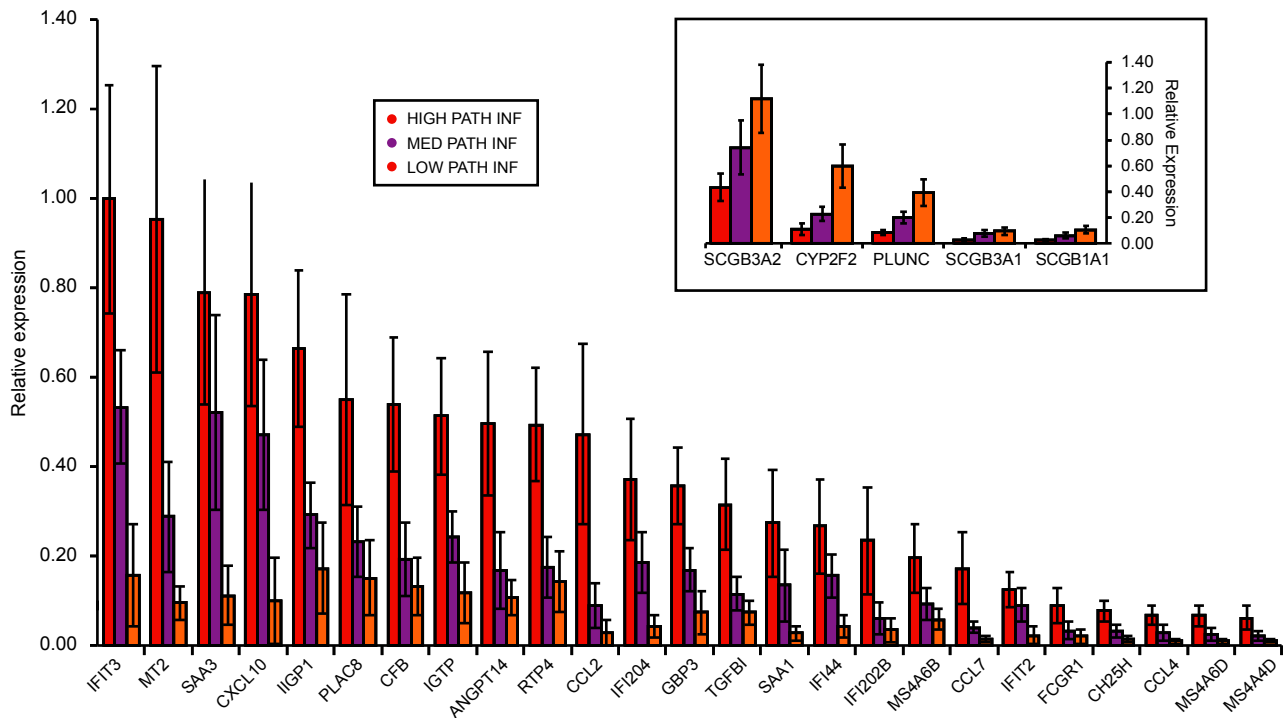

Supplement: Additional file 7 — Figure S4. Expression levels of select analog signature genes in HPI, MPI, and LPI conditions. These genes met the criterion of being expressed from greatest to least or from least to greatest (inset) by pathogenicity. Error bars represent standard errors of the means across all HPI, MPI, or LPI conditions in the compendium. [file 1752-0509-5-202-S7.PDF]
