# Supplementary material for: A chemokine gene expression signature derived from meta-analysis predicts the pathogenicity of viral respiratory infections
Source: BMC Syst Biol. 2011 Dec 22;5:202. doi: 10.1186/1752-0509-5-202 (PMC3297540; doi:10.1186/1752-0509-5-202)
Supplement: Additional file 3 — Table S1. Digital signature genes by Fisher's summary-statistic. [file 1752-0509-5-202-S3.DOC]

| **Gene Symbol** | **RefSeq ID** | **Dir HPI*** | **Gene Symbol** | **RefSeq ID** | **Dir HPI*** |
| --- | --- | --- | --- | --- | --- |
| B2M | NM_009735 | Up | SP100 | NM_013673 | Up |
| BIRC3 | NM_007464 | Up | SPP1 | NM_009263 | Up |
| C1QA | NM_007572 | Up | TCIRG1 | NM_016921 | Up |
| CCL5 | NM_013653 | Up | TNFAIP2 | NM_009396 | Up |
| CCR2 | NM_009915 | Up | TNFRSF12A | NM_013749 | Up |
| CD274 | NM_021893 | Up | TRIM30 | NM_009099 | Up |
| CEBPD | NM_007679 | Up | TUBB6 | NM_026473 | Up |
| CH25H | NM_009890 | Up | BEX2 | NM_009749 | Down |
| CLEC4D | NM_010819 | Up | CCKAR | NM_009827 | Down |
| CTPS | NM_016748 | Up | CES1 | NM_021456 | Down |
| CYP7B1 | NM_007825 | Up | CLDN10A | NM_021386 | Down |
| F3 | NM_010171 | Up | CRIP2 | NM_024223 | Down |
| FCGR2B | NM_010187 | Up | CYP1A1 | NM_009992 | Down |
| GADD45G | NM_011817 | Up | CYP2A4 | NM_009997 | Down |
| HCK | NM_010407 | Up | CYP2F2 | NM_007817 | Down |
| IFIT2 | NM_008332 | Up | DCXR | NM_026428 | Down |
| IL10RA | NM_008348 | Up | ELF5 | NM_010125 | Down |
| IL1B | NM_008361 | Up | GRB7 | NM_010346 | Down |
| IRF9 | NM_008394 | Up | GSTA3 | NM_010356 | Down |
| JUNB | NM_008416 | Up | HEY1 | NM_010423 | Down |
| LAP3 | NM_024434 | Up | HPN | NM_008281 | Down |
| LGMN | NM_011175 | Up | KCNMB4 | NM_021452 | Down |
| LITAF | NM_019980 | Up | MGAT3 | NM_010795 | Down |
| MMP3 | NM_010809 | Up | MGST1 | NM_019946 | Down |
| MS4A4D | NM_025658 | Up | MMP15 | NM_008609 | Down |
| NFKBIA | NM_010907 | Up | PCX | NM_008797 | Down |
| NNMT | NM_010924 | Up | PIGR | NM_011082 | Down |
| PARP9 | NM_030253 | Up | POR | NM_008898 | Down |
| PHC2 | NM_018774 | Up | S100G | NM_009789 | Down |
| PNP | NM_013632 | Up | SCNN1B | NM_011325 | Down |
| PPA1 | NM_026438 | Up | SERPINF1 | NM_011340 | Down |
| PSME1 | NM_011189 | Up | SFTPA1 | NM_023134 | Down |
| RAC2 | NM_009008 | Up | SFTPD | NM_009160 | Down |
| SELPLG | NM_009151 | Up | SLC34A2 | NM_011402 | Down |
| SEPX1 | NM_013759 | Up | TCF21 | NM_011545 | Down |
| SLFN2 | NM_011408 | Up | THRSP | NM_009381 | Down |
| SOCS3 | NM_007707 | Up | VPREB3 | NM_009514 | Down |

* “Dir HPI” refers to directionality of expression change (where “up” refers to HPI-up / LPI-down and “down” to HPI-down / LPI-up)

**Additional File 3. Table S1. Digital signature genes by Fisher’s summary-statistic**
