# Supplementary material for: A chemokine gene expression signature derived from meta-analysis predicts the pathogenicity of viral respiratory infections
Source: BMC Syst Biol. 2011 Dec 22;5:202. doi: 10.1186/1752-0509-5-202 (PMC3297540; doi:10.1186/1752-0509-5-202)
Supplement: Additional file 4 — Table S2. Digital signature genes by module-mapping. [file 1752-0509-5-202-S4.DOC]

| **Gene Symbol** | **RefSeq ID** | **Gene Symbol** | **RefSeq ID** | **Gene Symbol** | **RefSeq ID** |
| --- | --- | --- | --- | --- | --- |
| ABCB4 | NM_008830 | CCRN4L | NM_009834 | FABP1 | NM_017399 |
| ACTN2 | NM_033268 | CD28 | NM_007642 | FABP3 | NM_010174 |
| ACTN3 | NM_013456 | CD5L | NM_009690 | FABP4 | NM_024406 |
| ACVR1 | NM_007394 | CD8A | NM_001081110 | FASN | NM_007988 |
| ADAMTS1 | NM_009621 | CDC25B | NM_023117 | FCGR1 | NM_010186 |
| ADCY1 | NM_009622 | CDK1 | NM_007659 | FCGR2B | NM_010187 |
| ADIPOQ | NM_009605 | CDKN1A | NM_007669 | FCGR3 | NM_010188 |
| AFP | NM_007423 | CDKN1B | NM_009875 | FGFR3 | NM_008010 |
| AHSG | NM_013465 | CELSR1 | NM_009886 | FMO3 | NM_008030 |
| ALAS1 | NM_020559 | CENPA | NM_007681 | FOS | NM_010234 |
| ALAS2 | NM_009653 | CFD | NM_013459 | FOXC1 | NM_008592 |
| ALDH1A1 | NM_013467 | CFTR | NM_021050 | FOXC2 | NM_013519 |
| ALPL | NM_007431 | CHI3L3 | NM_009892 | GAB1 | NM_021356 |
| AMBP | NM_007443 | CHIA | NM_023186 | GADD45G | NM_011817 |
| ANGPTL3 | NM_013913 | CKS2 | NM_025415 | GALNT6 | NM_172451 |
| AP1M2 | NM_009678 | CLIC4 | NM_013885 | GALNTL2 | NM_030166 |
| APOA2 | NM_013474 | COL4A2 | NM_009932 | GCLC | NM_010295 |
| APOA5 | NM_080434 | CSRP3 | NM_013808 | GFPT1 | NM_013528 |
| AQP2 | NM_009699 | CST8 | NM_009978 | GJB3 | NM_008126 |
| ATP2A2 | NM_009722 | CYP1B1 | NM_009994 | GLI3 | NM_008130 |
| ATP2B2 | NM_009723 | DBP | NM_016974 | GPX2 | NM_030677 |
| AZGP1 | NM_013478 | DCXR | NM_026428 | GRIA1 | NM_008165 |
| B2M | NM_009735 | DES | NM_010043 | GRIN1 | NM_008169 |
| BBS2 | NM_026116 | DLX5 | NM_010056 | GRIN2B | NM_008171 |
| BC117090 | NM_001001332 | DMC1 | NM_010059 | GRM1 | NM_016976 |
| BCL3 | NM_033601 | DNAJB3 | NM_008299 | H1F0 | NM_008197 |
| BIRC5 | NM_009689 | DOC2G | NM_021791 | H2AFX | NM_010436 |
| BMP2 | NM_007553 | DSC1 | NM_013504 | HBEGF | NM_010415 |
| BRCA2 | NM_009765 | DSG2 | NM_007883 | HC | NM_010406 |
| C3 | NM_009778 | DSTN | NM_019771 | HDAC7 | NM_019572 |
| C3AR1 | NM_009779 | DYNLRB2 | NM_029297 | HIST1H1B | NM_020034 |
| C8G | NM_027062 | EDN1 | NM_010104 | HLF | NM_172563 |
| CALCA | NM_007587 | EDNRB | NM_007904 | HMGCS2 | NM_008256 |
| CAR13 | NM_024495 | EEA1 | NM_001001932 | HOXA2 | NM_010451 |
| CAR3 | NM_007606 | EGF | NM_010113 | HSF4 | NM_011939 |
| CAR8 | NM_007592 | EGR2 | NM_010118 | IAPP | NM_010491 |
| CASQ2 | NM_009814 | EHHADH | NM_023737 | IFI204 | NM_008329 |
| CCKAR | NM_009827 | EMR4 | NM_139138 | IGF1 | NM_010512 |
| CCL7 | NM_013654 | ENO3 | NM_007933 | IL10 | NM_010548 |
| CCL8 | NM_021443 | EPB4.1 | NM_183428 | IL12B | NM_008352 |
| CCNA1 | NM_007628 | EPHX1 | NM_010145 | IL1A | NM_010554 |
| CCNB1 | NM_172301 | EPHX2 | NM_007940 | IL1B | NM_008361 |
| CCR2 | NM_009915 | F3 | NM_010171 | IL2RB | NM_008368 |
| **Gene Symbol** | **RefSeq ID** | **Gene Symbol** | **RefSeq ID** | **Gene Symbol** | **RefSeq ID** |
| IL2RG | NM_013563 | PAX2 | NM_011037 | SLC28A2 | NM_172980 |
| IMPA1 | NM_018864 | PECAM1 | NM_008816 | SLC38A3 | NM_023805 |
| INHBB | NM_008381 | PER1 | NM_011065 | SLC43A1 | NM_001081349 |
| INPP1 | NM_008384 | PER2 | NM_011066 | SLC4A1 | NM_011403 |
| ITGB1BP2 | NM_013712 | PIK3C2G | NM_207683 | SLC6A13 | NM_144512 |
| ITGB3 | NM_016780 | PKP1 | NM_019645 | SLC6A18 | NM_001040692 |
| JAG1 | NM_013822 | PLCB1 | NM_019677 | SLC6A3 | NM_010020 |
| KCNJ11 | NM_010602 | PLG | NM_008877 | SLC7A5 | NM_011404 |
| KCNJ14 | NM_145963 | PLP1 | NM_011123 | SLC7A8 | NM_016972 |
| KCNK2 | NM_010607 | PON1 | NM_011134 | SMARCAD1 | NM_007958 |
| KCNMB2 | NM_028231 | POU5F1 | NM_013633 | SNCA | NM_009221 |
| KCNMB4 | NM_021452 | PPARA | NM_011144 | SOCS1 | NM_009896 |
| KDM4B | NM_172132 | PPARGC1A | NM_008904 | SOCS3 | NM_007707 |
| KIF1B | NM_008441 | PRKDC | NM_011159 | SPAG5 | NM_017407 |
| KIF5B | NM_008448 | PROK2 | NM_015768 | SPNA1 | NM_011465 |
| KLF9 | NM_010638 | PTGDS | NM_008963 | SPNB2 | NM_009260 |
| KRT10 | NM_010660 | PTGER3 | NM_011196 | SPNB2 | NM_175836 |
| KRT14 | NM_016958 | PTGER4 | NM_008965 | SPP1 | NM_009263 |
| KRT4 | NM_008475 | PTGS2 | NM_011198 | SPRR2A1 | NM_011468 |
| LRP11 | NM_172784 | PTPRC | NM_011210 | SRRT | NM_031405 |
| LRP5 | NM_008513 | PTTG1 | NM_013917 | ST8SIA2 | NM_009181 |
| LRP6 | NM_008514 | PYGM | NM_011224 | STAR | NM_011485 |
| MAFB | NM_010658 | RAMP1 | NM_016894 | STAT5A | NM_011488 |
| MAPT | NM_010838 | RBP4 | NM_011255 | STFA3 | NM_025288 |
| MARCO | NM_010766 | RBPJ | NM_009035 | STS | NM_009293 |
| MEFV | NM_019453 | RCAN3 | NM_022980 | SYT10 | NM_018803 |
| MSR1 | NM_031195 | RELN | NM_011261 | SYT6 | NM_018800 |
| MST1 | NM_008243 | REM1 | NM_009047 | SYTL2 | NM_001040087 |
| MT1 | NM_013602 | RGR | NM_021340 | TACC3 | NM_001040435 |
| MT2 | NM_008630 | S100A9 | NM_009114 | TBX1 | NM_011532 |
| MYC | NM_010849 | S100B | NM_009115 | TBX5 | NM_011537 |
| MYH6 | NM_010856 | SCD2 | NM_009128 | TEF | NM_153484 |
| MYL7 | NM_022879 | SCGB1A1 | NM_011681 | TEKT2 | NM_011902 |
| MYO1B | NM_010863 | SEC14L2 | NM_144520 | TGM3 | NM_009374 |
| NAT3 | NM_008674 | SEMA3F | NM_011349 | TLR1 | NM_030682 |
| NCF1 | NM_010876 | SEMA6A | NM_018744 | TLR3 | NM_126166 |
| NEDD4 | NM_010890 | SERPINA6 | NM_007618 | TNFRSF12A | NM_013749 |
| NEDD9 | NM_017464 | SERPINC1 | NM_080844 | TNN | NM_177839 |
| NPR1 | NM_008727 | SFTPA1 | NM_023134 | TNNI3 | NM_009406 |
| NRTN | NM_008738 | SFTPC | NM_011359 | TNNT2 | NM_011619 |
| NUMB | NM_010949 | SFTPD | NM_009160 | TRP63 | NM_011641 |
| OLFR476 | NM_146924 | SGCG | NM_011892 | TRPC6 | NM_013838 |
| OXR1 | NM_130885 | SLC25A22 | NM_026646 | TNNI3 | NM_009406 |

| **Gene Symbol** | **RefSeq ID** | **Gene Symbol** | **RefSeq ID** | **Gene Symbol** | **RefSeq ID** |
| --- | --- | --- | --- | --- | --- |
| TXNRD1 | NM_015762 | WISP1 | NM_018865 | ZFP36 | NM_011756 |
| UBE2C | NM_026785 | XRCC6 | NM_010247 |  | XM_988516 |
| VTN | NM_011707 | ZBTB7A | NM_010731 |  |  |

Note that overall expression of this module (Module 5) was found to be down-regulated in HPI and up-regulated in LPI in Genomica.

**Additional File 4. Table S2. Digital signature genes by module-mapping**
