# Supplementary material for: A chemokine gene expression signature derived from meta-analysis predicts the pathogenicity of viral respiratory infections
Source: BMC Syst Biol. 2011 Dec 22;5:202. doi: 10.1186/1752-0509-5-202 (PMC3297540; doi:10.1186/1752-0509-5-202)
Supplement: Additional file 5 — Table S3. Analog signature genes by fold change-based z-test. [file 1752-0509-5-202-S5.DOC]

| **Gene Symbol** | **RefSeq ID** | **Dir HPI*** | **Gene Symbol** | **RefSeq ID** | **Dir HPI*** |
| --- | --- | --- | --- | --- | --- |
| ANGPTL4 | NM_020581 | Up | SLFN3 | NM_011409 | Up |
| CCL2 | NM_011333 | Up | SLFN4 | NM_011410 | Up |
| CCL4 | NM_013652 | Up | TGFBI | NM_009369 | Up |
| CCL7 | NM_013654 | Up | AHCYL1 | NM_145542 | Down |
| CFB | NM_008198 | Up | AOX3 | NM_023617 | Down |
| CH25H | NM_009890 | Up | CCKAR | NM_009827 | Down |
| CXCL10 | NM_021274 | Up | CLDN10A | NM_021386 | Down |
| CXCL11 | NM_019494 | Up | CXADR | NM_009988 | Down |
| CXCL9 | NM_008599 | Up | CYP1A1 | NM_009992 | Down |
| FCGR1 | NM_010186 | Up | CYP2F2 | NM_007817 | Down |
| GBP3 | NM_018734 | Up | CYP4F15 | NM_134127 | Down |
| IFI202B | NM_011940 | Up | DLX3 | NM_010055 | Down |
| IFI204 | NM_008329 | Up | HK1 | NM_010438 | Down |
| IFI44 | NM_133871 | Up | LIMD2 | NM_172397 | Down |
| IFIT2 | NM_008332 | Up | MATN4 | NM_013592 | Down |
| IFIT3 | NM_010501 | Up | MCHR1 | NM_145132 | Down |
| IGTP | NM_018738 | Up | MUC5B | NM_028801 | Down |
| IIGP1 | NM_021792 | Up | MYO1C | NM_008659 | Down |
| MMP8 | NM_008611 | Up | PLUNC | NM_011126 | Down |
| MS4A4D | NM_025658 | Up | PON1 | NM_011134 | Down |
| MS4A6B | NM_027209 | Up | S100G | NM_009789 | Down |
| MS4A6D | NM_026835 | Up | SCGB1A1 | NM_011681 | Down |
| MT2 | NM_008630 | Up | SCGB3A1 | NM_170727 | Down |
| PLA1A | NM_134102 | Up | SCGB3A2 | NM_054038 | Down |
| PLAC8 | NM_139198 | Up | SYT10 | NM_018803 | Down |
| RTP4 | NM_023386 | Up | TPPP3 | NM_026481 | Down |
| SAA1 | NM_009117 | Up | UGT2B34 | NM_153598 | Down |
| SAA2 | NM_011314 | Up | VEGFB | NM_011697 | Down |
| SAA3 | NM_011315 | Up |  |  |  |

* “Dir HPI” refers to directionality of expression change in HPI compared to LPI.

**Additional File 5. Table S3. Analog signature genes by fold change-based *z*-test**
