# Supplementary material for: A chemokine gene expression signature derived from meta-analysis predicts the pathogenicity of viral respiratory infections
Source: BMC Syst Biol. 2011 Dec 22;5:202. doi: 10.1186/1752-0509-5-202 (PMC3297540; doi:10.1186/1752-0509-5-202)
Supplement: Additional file 6 — Figure S3. Characterization of the 74-gene digital signature of pathogenicity by networks of known interactions. Genes present in the signature are indicated in gray shapes. (A) For the 44-gene subset up-regulated in HPIs and down-regulated in LPIs. (B) For the 30-gene subset down-regulated in HPIs and up-regulated in LPIs. [file 1752-0509-5-202-S6.PDF]

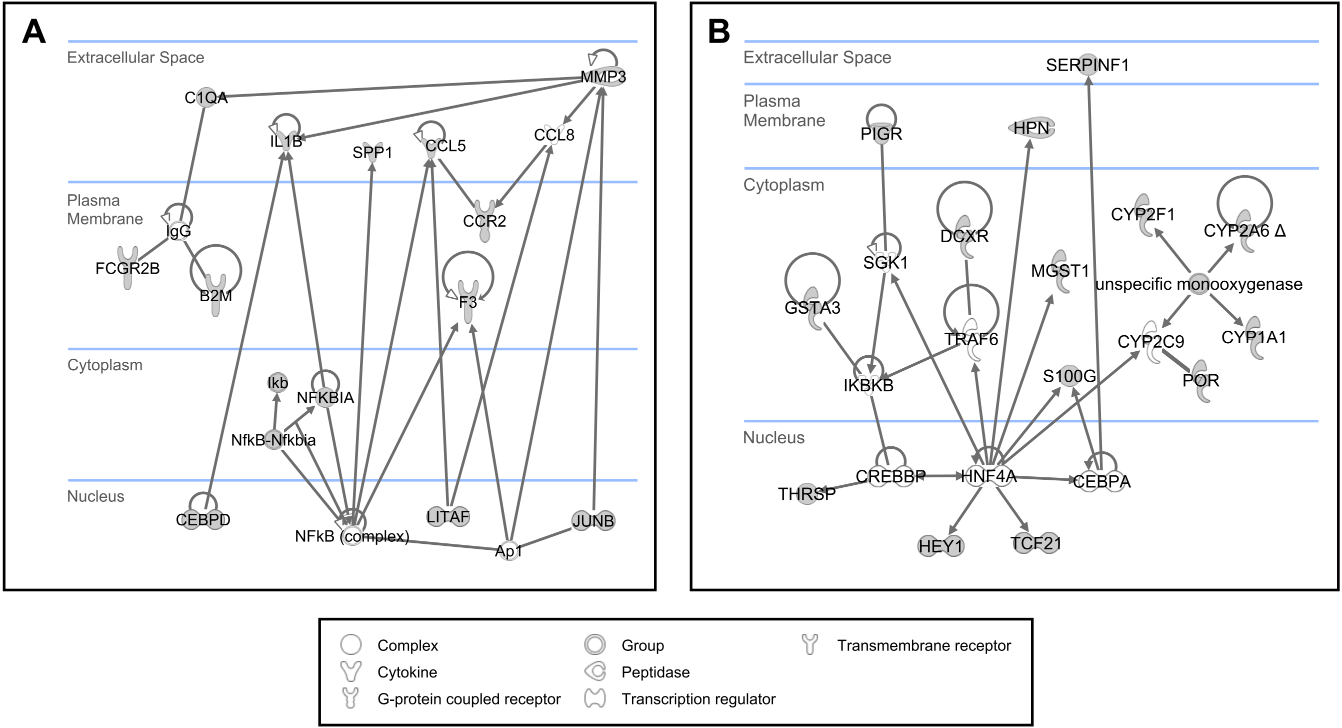
A
Extracellular Space
Plasma Membrane
Cytoplasm
Nucleus
MMP3
C1QA
IL1B
SPP1
CCL5
CCL8
CCR2
IgG
FCGR2B
B2M
F3
Ikb
NFKBIA
NfkB-Nfkbia
CEBPD
NFkB (complex)
LITAF
JUNB
Ap1
B
Extracellular Space
SERPINF1
Plasma Membrane
PIGR
HPN
Cytoplasm
CYP2F1
CYP2A6 Δ
DCXR
SGK1
MGST1
unspecific monooxygenase
GSTA3
TRAF6
CYP2C9
CYP1A1
IKBKB
S100G
POR
Nucleus
CREBBP
HNF4A
CEBPA
THRSP
HEY1
TCF21
Complex
Cytokine
G-protein coupled receptor
Group
Peptidase
Transcription regulator
Transmembrane receptor
